# 

**Published:** 1902-02-01

**Authors:** 


### NOTICE TO CORRESPONDENTS.

All MSS., Letters, Books for Review, and other matters intended for the Editor should be addressed THE EDITOR, "THE HOSPITAL," THE HOSPITAL BUILDING, 28 & 29 SOUTHAMPTON STREET, STRAND, W.C.

The Editor cannot undertake to return rejected MS., even when accompanied by stamped directed envelope.

**Notice to Advertisers and Subscribers.**

All Advertisements, Orders for Copies of THE HOSPITAL, and Business Communications should be addressed to **THE MANAGER** (**Not to the Editor**), THE HOSPITAL, 28 & 29 Southampton Street, Strand, London, W.C.

The Cost of Subscription, post free, is as follows:—Per week, 2½d.; per quarter, 2s. 8d.; per half-year, 5s. 3d.; per annum, 10s. 6d. Foreign:—Per annum, 15s. 2d., payable, in all cases, in advance.

**NOTICE.—Vol. XXX. of "The Hospital" (April 1901, to September 1901), in handsome cloth binding can now be obtained at the office of this Journal, price, 7s. 6d. Cases for binding the Numbers contained in this Volume 1s. 6d. Index to Vol. XXX., 2½d., post free.**

**The Monthly Part for February is now ready. Price 6d., by post 8d.**

### BOOKS RECEIVED.

Scientific Press.

"Syllabus of Lectures to Nurses." By Andrew Davidson, M.D.

Edward Lloyd.
"The London Manual for 1902." Edited by Robert Donald.

Edward Arnold.

"Human Embryology and Morphology." By Arthur Keith, M.D., F.R.C.S.

John Wright and Co.
"Elementary Bandaging and Surgical Dressing." By the late Walter Pye, F.R.C.S., revised by Thos. Carwardine, M.S., F.R.C.S.

Century Printing Company.
"The War against Consumption." By Dennis Vinrace, M.R.C.S., L.S.A., revised by John H. Vinrace, M.B., M.R.C.P.

Baillière, Tindall and Cox.
"The Imperial Health Manual." Edited by Anthony Roche, M.R.C.P.I.

William Wood and Co.
"Vaginal Cancer." By W. Roger Williams, F.R.C.S.

James Willing, Junior.
"Willing's Press Guide, 1902."

### SCRAPS AND GLEANINGS.

THE Fishmongers' Company has contributed an annual subscription of £1,000 to King Edward's Hospital Fund.

AT a public meeting held at the Leatherhead Institute early in December, subscriptions to the amount of £475 were promised towards the cost of building a new cottage hospital.

FIVE hundred women of Aldershot Camp have presented Lady Audrey Buller with a life-governorship of the Aldershot Hospital as a mark of their appreciation of all that her ladyship did for them during their husbands' absence in South Africa.

FIVE hundred pounds per annum is required to free the Victoria Hospital, Folkestone, from debt, and the secretary in appealing for funds states that 400 in-patients and 1,600 out-patients are treated annually, and the hospital is only endowed to the extent of £300 per annum.

HIS MAJESTY THE KING has kindly sent 10 brace of pheasants to the National Orthopædic Hospital, 34 Great Portland Street, W. The same hospital recently received from its President, the Duke of Marlborough, a large box of toys, etc., for the patients at Christmas time.

AT the special meeting of the governors of the General Infirmary at Gloucester, details were given of a nurses' home which it was proposed to erect, the approximate cost of which would be £6,000, exclusive of lighting, heating, etc. The additional cost of a new hot-water service which they had decided to put in would amount, it was stated, to about £2,500.

### THE STUDENT OF MEDICINE.

### APPOINTMENTS.

J. L. Adam, M.B., C.M.Aberd., appointed District Medical Officer of the Hartley Wintney Union. A. P. Beddard, M.D., M.R.C.P., appointed Physician to the West London Hospital.

**E. H. Beresford**, M.R.C.S., L.R.C.P.Lond., appointed Medical Superintendent of the Tooting Bec Asylum. **Graham T. B. Blick**, L.R.C.P.Lond., M.R.C.S., L.S.A., appointed Medical Officer in charge of Broome Hospital. **Walter Broadbent**, M.A., M.D.Cantab., L.R.C.P.Lond., appointed Assistant Physician to the Sussex County Hospital. **Harry L. Brownlow**, F.R.C.S.Eng., L.R.C.P., appointed Assistant Surgeon to the Royal United Hospital, Bath. **Frederick S. Butler**, M.B.Melb., appointed District Medical Officer and Public Vaccinator for Lawler, West Australia. **R. F. Castle**, M.B., B.C.Cantab., appointed District Medical Officer of the Barnsley Union. **W. F. Cholmeley**, F.R.C.S., appointed Honorary Assistant Surgeon to the Wolverhampton and South Staffordshire General Hospital. **R. Veitch Clark**, M.A., B.Sc., M.B., Ch.B.Edin., appointed Assistant in the Physiological Department, Yorkshire College, Victoria University, Leeds. **H. Spencer Cooper**, L.R.C.P.Lond., M.R.C.S.Eng., L.S.A., appointed Medical Officer and Public Vaccinator for the Yaxley District of the Peterborough Union. **E. W. Deane**, M.B., Ch.B.Melb., appointed Officer of Health for the Borough of Carisbrook, Victoria. **Howard Dent**, M.B.Durh., F.R.C.S.Eng., appointed Assistant Surgeon to the Wolverhampton and Staffordshire General Hospital. **Edwin Loudney**, M.R.C.S.Eng., L.R.C.P.Edin., appointed Government Medical Officer and Vaccinator at Port Macquarie, New South Wales. **A. M. Erskine**, M.B., D.P.H., appointed Medical Officer of Health to the Goole Urban District Council and Medical Superintendent of the Fever Hospital **Richard H. J. Fetherston**, M.D.Edin., L.R.C.S.I., appointed Certifying Factory Surgeon for the Metropolitan District of Melbourne. **A. Freear**, M.R.C.S., L.R.C.P.Lond., appointed Resident Medical Officer to the Medway Union Workhouse. **H. M. Guest**, M.R.C.S., L.R.C.P.Lond., appointed District Medical Officer of the Alcham Union. **P. G. Garrett**, M.B., B.S.Durh., appointed District Medical Officer for the Hinckley Union. **Arthur M. Jackson**, M.D.Oxon, appointed Medical Superintendent of the Notts County Asylum. **Frederick Lace**, F.R.C.S., appointed Surgeon to the Royal United Hospital, Bath. **J. O. Littlewood**, M.R.C.S., L.R.C.P.Lond., D.P.H., appointed Visiting Medical Officer to the Nottinghamshire Sanatorium for Treatment of Consumption, Sherwood Forest. **Thos. J. Lovergan**, M.R.C.P., L.R.C.S.Edin., appointed District Medical Officer and Public Vaccinator for Northampton, West Australia. **Richard A. A. Manly**, M.B.Melb., appointed Medical Officer of Health for the Shire of Lancefield, Victoria. **H. Maturin**, L.R.C.P.Edin., M.R.C.S.Eng., appointed Medical Officer of Health for the Hartley Wintney Rural District. **S. H. Perry**, M.D.Lond., appointed Medical Officer of Health to the Spalding Rural District Council. **W. L. Pethybridge**, M.D., B.Sc.Lond., M.R.C.S., L.R.C.P., appointed Honorary Anæsthetist to the Royal Eye Infirmary, Plymouth. **John Howson Ray**, Ch.M.Vict., F.R.C.S.Eng., appointed Surgeon to the Manchester Children's Hospital. **Thomas Sayer**, M.R.C.S.Eng., L.R.C.P.Lond., has been appointed Resident Medical Officer at the Leeds Sanatorium for Consumptives at Gateforth. **H. R. D. Spitta**, M.B., B.S.Durh., M.R.C.S., L.R.C.P., D.P.H.Camb., Assistant Lecturer on Bacteriology in St. George's Hospital Medical School, appointed Assistant Lecturer on Public Health. **William Atkin Thompson**, M.R.C.S.Eng., L.R.C.P.Lond., appointed House Surgeon to the Rochdale Infirmary. **F. W. Twort**, M.R.C.S., L.R.C.P.Lond., appointed Assistant Bacteriologist to the London Hospital and Medical College. **C. Edward Wallis**, M.R.C.S., L.R.C.P., L.D.S., appointed Dental Surgeon to Rochester House Asylum under the Metropolitan Asylums Board.

